# Correction: A low-cost liquid crystal display (LCD)-printed microfluidic device: fabrication and application in the biodegradation of petroleum hydrocarbons in petroleum refinery sludge

**DOI:** 10.1039/d6ra90054b

**Published:** 2026-05-28

**Authors:** Amlan Ashish, Ankita Debnath, Poulomi Biswas, Ramkrishna Sen, Gorachand Dutta

**Affiliations:** a NanoBiosensors and Biodevices Lab, School of Medical Sciences and Technology, Indian Institute of Technology Kharagpur Kharagpur-721302 India g.dutta@smst.iitkgp.ac.in; b Advanced Technology Development Centre (ATDC), Indian Institute of Technology Kharagpur Kharagpur-721302 India; c Department of Bioscience and Biotechnology, Institute of Technology Kharagpur Kharagpur-721302 India; d ALS Testing Services India Pvt Ltd Hyderabad India

## Abstract

Correction for ‘A low-cost liquid crystal display (LCD)-printed microfluidic device: fabrication and application in the biodegradation of petroleum hydrocarbons in petroleum refinery sludge’ by Amlan Ashish *et al.*, *RSC Adv.*, 2026, **16**, 23044–23057, https://doi.org/10.1039/D5RA06174A.

The authors regret that the author contributions were shown incorrectly in the original article. Amlan Ashish, Ankita Debnath and Poulomi Biswas all contributed equally to this work. The corrected author contributions are as shown above.

The Royal Society of Chemistry apologises for these errors and any consequent inconvenience to authors and readers.

